# Development of a Collagen Fibre Remodelling Rupture Risk Metric for Potentially Vulnerable Carotid Artery Atherosclerotic Plaques

**DOI:** 10.3389/fphys.2021.718470

**Published:** 2021-10-29

**Authors:** Milad Ghasemi, Robert D. Johnston, Caitríona Lally

**Affiliations:** ^1^Trinity Centre for Biomedical Engineering, Trinity College Dublin, Dublin, Ireland; ^2^Department of Mechanical, Manufacturing and Biomedical Engineering, School of Engineering, Trinity College Dublin, Dublin, Ireland; ^3^Advanced Materials and Bioengineering Research Centre (AMBER), Royal College of Surgeons in Ireland and Trinity College Dublin, Dublin, Ireland

**Keywords:** plaque rupture, atherosclerosis, carotid arteries, finite element, arterial remodelling

## Abstract

Atherosclerotic plaque rupture in carotid arteries can lead to stroke which is one of the leading causes of death or disability worldwide. The accumulation of atherosclerotic plaque in an artery changes the mechanical properties of the vessel. Whilst healthy arteries can continuously adapt to mechanical loads by remodelling their internal structure, particularly the load-bearing collagen fibres, diseased vessels may have limited remodelling capabilities. In this study, a local stress modulated remodelling algorithm is proposed to explore the mechanical response of arterial tissue to the remodelling of collagen fibres. This stress driven remodelling algorithm is used to predict the optimum distribution of fibres in healthy and diseased human carotid bifurcations obtained using Magnetic Resonance Imaging (MRI). In the models, healthy geometries were segmented into two layers: media and adventitia and diseased into four components: adventitia, media, plaque atheroma and lipid pool (when present in the MRI images). A novel meshing technique for hexahedral meshing of these geometries is also demonstrated. Using the remodelling algorithm, the optimum fibre patterns in various patient specific plaques are identified and the role that deviations from these fibre configurations in plaque vulnerability is shown. This study provides critical insights into the collagen fibre patterns required in carotid artery and plaque tissue to maintain plaque stability.

## Introduction

Atherosclerotic plaque rupture in carotid arteries can lead to stroke which is one of the leading causes of death worldwide (Benjamin et al., 2017). The accumulation of atherosclerotic plaque in an artery changes the mechanical properties of the vessel (Holzapfel et al., 2000). Healthy arteries continuously adapt to mechanical loads by remodelling their internal structure (Driessen et al., 2008). Remodelling is a complex process which occurs in biological tissues in response to alterations in their mechanical, chemical or biological environment (Rouillard and Holmes, 2012; Aparício et al., 2016). Gibbons and Dzau (1994) described the remodelling process as the ability of the tissue to adapt in the following ways; **(i)** cell growth, **(ii)** cell death, **(iii)** cell migration, and **(iv)** production or degradation of extracellular matrix such as elastin and collagen fibres (Gibbons and Dzau, 1994).

Collagen fibres are the main load bearing constituent in arterial tissue and many other soft biological tissues (deBotton and Hariton, 2006; Baaijens et al., 2010). The important role of collagen fibres in bearing physiological loads is emphasised in studies such as Sommer et al. (2010) and Gaul et al. (2020). The specific role of collagen fibres in arteries under supra-physiological loads is discussed in studies such as Weisbecker et al. (2013), Schriefl et al. (2015), and Ghasemi et al. (2018).

The anisotropic response of the arterial wall is predominantly associated with the distribution of the collagen fibres in the tissue and many studies have demonstrated the need for structural constitutive models to incorporate the orientation and distribution of fibres to more accurately capture the mechanical response of the tissue (Holzapfel et al., 2000; Gasser et al., 2006). In arteries, the distribution of collagen fibres varies through the wall thickness where fibres are more aligned at the innermost layer of the arterial wall and more dispersed in the outermost layer (adventitial layer), establishing a well-known helical structure of fibres in the vessel wall (Driessen et al., 2005a; Hariton et al., 2007).

Collagen is also a prominent constituent of atherosclerotic plaques (Rekhter et al., 1993). Chai et al. (2015) measured the local anisotropic mechanical response of different components of atherosclerotic plaque by performing indentation tests at large strain levels (Chai et al., 2015). Chai et al. (2015) concluded that different components of atherosclerotic plaque show different levels of anisotropic behaviour. In that study, the level of anisotropy in the mechanical response of the tissue was found to be related to the stiffness and distribution of collagen fibres in the plaque (Chai et al., 2015). Pagiatakis et al. (2015) compared the actual measured orientation of fibres in fibrous plaque caps, obtained from human coronary atherosclerotic plaques, with the direction of the maximum principal stresses calculated using fluid-structure interaction computational models. They observed that the principal stresses were oriented circumferentially in the healthy tissue and more axially in the fibrous cap (Pagiatakis et al., 2015). Using Diffusion Tensor Imaging (DTI), Akyildiz et al. (2017) characterised the 3-D orientation of fibres in carotid atherosclerotic plaques and showed that the distribution of fibres varies in different parts of the plaque (Akyildiz et al., 2017), whilst Douglas et al. (2017) explored the fibre distributions in coronary atherosclerotic plaques (Douglas et al., 2017). In that study, Douglas et al. (2017) extracted data from histological images to define material parameters for angle and dispersion of collagen fibres in the constitutive model proposed by Gasser et al. (2006). They also investigated the values of stress measured using these material properties as a clinical measure for assessing the risk of plaque rupture (Douglas et al., 2017).

Whilst the critical role of collagen fibres orientation is emphasised in the aforementioned studies, to the best of the authors’ of knowledge, no study to-date has been able to directly link the risk of plaque rupture with a lack of collagen fibre remodelling to the optimum fibre configuration.

Remodelling of collagen fibres in the healthy arterial wall has been the focus of many studies (Hariton et al., 2007; Driessen et al., 2008; Creane et al., 2010; Fausten et al., 2016). Whilst it is known that collagen fibres remodel in the arterial wall to maximise the load bearing capacity of the tissue (Baaijens et al., 2010), a number of different forms of mechanical stimulus have been proposed to calculate the optimum distribution of collagen fibres which would give the maximum load bearing capacity to the tissue.

Hariton et al. (2007) assumed that collagen fibres evolve toward the optimum distribution of fibres, determined from the ratio of the maximum and intermediate principal stresses (Hariton et al., 2007). Similar assumptions were made in studies conducted by Pagiatakis et al. (2015) and Fausten et al. (2016). The ratio of the two largest principal stretches were used in studies such as Driessen et al. (2005a, b), Grytz and Meschke (2010), and Creane et al. (2011) to calculate the optimum configuration of the fibres (Driessen et al., 2005a,b; Grytz and Meschke, 2010; Creane et al., 2012). Waffenschmidt and Menzel (2014) assumed that the direction of collagen fibres evolved to minimise total potential energy of the arterial tissue (Waffenschmidt and Menzel, 2014). This approach was compared with methods implemented in Hariton et al. (2007) in the research conducted by Qi et al. (2015). For further information on different remodelling algorithms implemented to capture the orientation of fibres in arteries, the reader is referred to, for example, Baaijens et al. (2010), Qi et al. (2015), and Fausten et al. (2016).

Whilst different forms of remodelling algorithms are suggested in the literature, only a limited number have been used to investigate the distribution of fibres in real healthy and diseased patient specific geometries. Creane et al. (2011) used a strain driven remodelling algorithm to explore fibre patterns in healthy and diseased carotid arteries obtained from CT imaging (Creane et al., 2011). The models predicted a helical distribution of collagen fibres in the non-branching regions of the bifurcations and a more complex distribution of fibres was obtained at the apex of the bifurcation and in regions of plaque burden, which altered the stress distribution in the artery. Fausten et al. (2016) used a stress driven remodelling algorithm to investigate the distribution of fibres in the geometry of a human common carotid (Fausten et al., 2016).

Recently, there has been investigations into the stiffness and strain levels of vulnerable plaques in atherosclerotic carotid arteries, using imaging techniques such as ultrasound elastography (Schaar et al., 2003; Huang et al., 2016). The results of these studies show that plaques with higher grades of vulnerability are softer and consequently experience higher strain levels. However, these studies do not provide any further insights into the influence of the remodelling of collagen fibres on the vulnerability of these atherosclerotic plaques.

Creane et al. (2011) proposed a remodelling metric which calculated the mean rotational effort required for one family of collagen fibres to re-orient into another distribution using an adaptation of the concept of the earth mover’s distance. It was shown that the proposed remodelling metric had higher values in the geometries obtained from symptomatic patients indicating that more effort was required for fibres in these arteries to reach their optimum configuration in comparison with arteries obtained from asymptomatic patients. Although the proposed remodelling metric was able to successfully distinguish the symptomatic and asymptomatic patients, it couldn’t provide enough insights into alterations in the mechanical behaviour of the plaque tissue during the remodelling process or how remodelling of fibres might potentially reduce the risk of plaque rupture, given that fibres were limited to the healthy arterial tissue.

In this study, a local stress modulated remodelling algorithm is proposed to explore the mechanical response of the tissue to the remodelling of collagen fibres. This stress driven remodelling algorithm is then used to predict the optimum distribution of fibres in healthy and diseased human carotid bifurcations obtained using Magnetic Resonance Imaging (MRI).

In an earlier study by the authors (Ghasemi et al., 2018), the influence of collagen fibres in the mechanical response of arterial tissue to physiological and supra-physiological loads was successfully captured using a continuum damage model (CDM) (Ghasemi et al., 2018). The current study uses this CDM as a means to develop a novel remodelling metric (RM) to characterise the lack of remodelling to the optimum collagen fibre distribution in atherosclerotic plaques. This enables the stiffness of arterial tissue to be correlated with the distribution of collagen fibres within the arterial wall. Arterial tissue should have its optimum stiffness and strength when collagen fibres are aligned with an optimum configuration measured according to the ratio of maximum to intermediate Cauchy stresses (Hariton et al., 2007; Creane et al., 2012; Fausten et al., 2016). However, the further away collagen fibres are from this optimum configuration, the more the stiffness of the tissue is reduced, and this could result in the higher strain levels observed in high-risk rupture prone atherosclerotic plaques (Schaar et al., 2003; Huang et al., 2016).

To fully assess the functionality of this remodelling metric, three different scenarios were postulated where fibres in the atherosclerotic plaque were assumed to be: (i) parallel to the direction of intermediate principal stress (ii) at 45^*o*^ with respect to the direction of the maximum principal stress and (iii) parallel to the direction of the maximum principal stress. In each case fibres were remodelled toward the optimum fibre distribution. Using CDM, the internal variables which were associated with the remodelling metric could evolve as the fibres remodelled toward the optimum fibre configuration. Larger remodelling values were obtained in cases when fibres were further away from their optimum configuration, thereby indicating that remodelling in the arterial wall and plaques was not optimised in these cases. This lack of remodelling results in a weaker arterial wall that could increase the risk of atherosclerotic plaque rupture. Consequently, this remodelling metric can be used to quantitatively link the vulnerability of atherosclerotic plaques to the spatial configuration of collagen fibres in the tissue. Such valuable information enhances the assessment of the risk of plaque rupture in asymptomatic and symptomatic carotid disease patients by investigating the distribution of collagen fibres in arterial tissue.

## Materials and Methods

### Imaging Protocol

*In vivo* MRI scans of carotid arteries were obtained from five patients under evaluation for a carotid endarterectomy procedure. A 3T whole body MRI scan (Achieva, Phillips Medical Systems, Best, Netherlands) combined with an 8-channel dedicated bilateral carotid artery coil (Shanghai Chenguang Medical Technologies, Shanghai, China) were used. The imaging parameters used for the creation of the geometries are given in Table 1 with the field of view (FOV) centred on the vessel bifurcation. To improve the scanning resolution, the procedure was repeated twice with a 1 mm offset in the z-direction giving our models an apparent slice thickness of 1 mm. The scanning of patients took place at the Advanced Centre of Medical Imaging (CAMI) in St. James Hospital in Dublin while the scanning of volunteers took place in the Trinity Centre for Neuroscience (TCIN). Ethical approval was obtained for the scanning of volunteers and patients by the relevant regulatory bodies.

**TABLE 1:** Scanning parameters used for the reconstruction of arteries.

Acquisition parameters	T1W 2D	T2W 2D	T2W_TSE 2D	TOF 3D
Resolution (mm)	0.5 × 0.5	0.5 × 0.5	0.5 × 0.5	0.5 × 0.5
Repetition time (TR) (ms)	984	3,000	2 R-R Intervals	25
Echo time (TE) (ms)	11	38	38	3
Slice thickness (mm)	3	3	3	3
Number of slices	8	8	8	48
Number of echoes	0	0	12	0

### Segmentation Protocol

In healthy geometries, arterial walls were manually segmented from MR T2 weighted images using Simpleware ScanIP (Synopsys, Inc., Mountain View, United States). The dataset was initially cropped to the region of interest and the vessel wall was delineated from MR T2 weighted Images. Using T2 weighted MR images enabled the variability in the wall thickness to be seen throughout the vessel wall as emphasised in Delfino et al., 1997.

To obtain the required information about the plaque components in the vessel wall, multiple image contrasts were used. Iso-intense to hyper-intense areas on MR T1W images with varying intensity on MR T2W and MR TOF images were considered to correspond to the lipid rich necrotic core (LRNC) similar to the methods used in Cai et al. (2005) and Saam et al. (2005). Furthermore, an MR T2-TSE sequence was used to isolate the plaque components (Biasiolli et al., 2013). This difference can be observed due to the different relaxation times attributed to each component.

### Geometry Preparation

#### Healthy and Diseased Carotid Bifurcation

**FIGURE 1:**
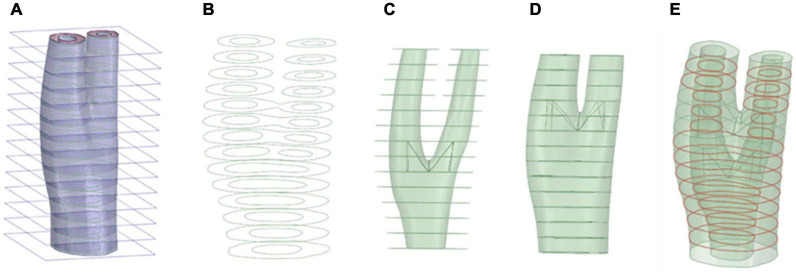
Geometry preparation steps. Sectioning the geometry and extraction of the curves **(A,B)**, respectively. **(C)** Skin the inner surface, **(D)** Skin the outer surface, **(E)** Stitching the geometry surfaces where required (red curve).

In order to avoid the sharp edges in the reconstructed artery, the segmented geometries needed to be smoothed. This was especially important at the bifurcations, where an un-smoothed geometry could result in distorted elements that would cause numerical convergence issues. Using ANSYS Spaceclaim (ANSYS Inc., United States), curves were extracted from the boundaries of different components of the vessel wall such as the lumen, plaque components and the outermost surface of the vessel walls by obtaining the cross section of the geometry by sectioning using a series of parallel planes (see Figure 1). These curves were then connected to construct the inner and outer surfaces of the arterial wall and plaque components. The geometry was stitched together to ensure there were no gaps between component interfaces in the next step. The blend function was then applied to connect the inner and outer vessel wall and ensure a watertight geometry. This process is illustrated in Figure 1. These smoothed surfaces were then saved as STL meshes to export to a finite element (FE) processor.

### Hexahedral Meshing of Bifurcations and Plaques

**FIGURE 2:**
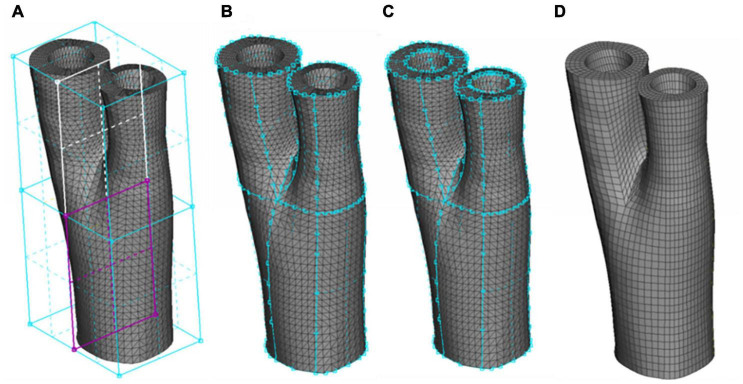
The Sequence of generating a structured hexahedral mesh from a human carotid bifurcation. **(A)** The STL mesh and definition of the boxes indicating the common, internal and external carotid artery. **(B)** Associating the boxes to the outermost surface of the STL mesh. **(C)** Using the O-Grid function to define the luminal surface. **(D)** Final meshed geometry.

The production of a high-quality mesh is an essential step for an accurate FE analysis, however, producing structured meshes is a major challenge for complex 3-D arterial geometries, particularly at bifurcations (Creane et al., 2010; De Santis et al., 2011). Development of a meshing protocol to accommodate the structured hexahedral meshes for 3-D real arterial geometries is a difficult task for most commercial FE packages (Creane et al., 2010). There are a few studies dealing with producing high quality meshes from real arterial geometries in the literature (Antiga et al., 2002; Zhang et al., 2007; Creane et al., 2010; De Santis et al., 2011; Tarjuelo-Gutierrez et al., 2014), however, most of these studies consider only luminal blood flow as the main 3D geometry and simply assign uniform wall thickness for the vessel wall. Here, a novel meshing technique is introduced to enable both the inner and outer layers of the arterial wall to be considered in the mesh geometry. Figure 2 presents a schematic of the different steps of our meshing protocol.

The STL meshes, constructed from smoothed geometries were hex meshed using ANSA (v17.0, BETA CAE Systems, Thessaloniki, Greece). Appling the *Hexa Block module*, the volume of the artery was defined initially in the form of a box. This box was then split into independent boxes defining the three sections of the carotid artery: common carotid artery, and internal and external branches of the carotid artery. These boxes converged at the apex of the bifurcation (see Figure 2A). The perimeters of each box were assigned to the outer wall of the geometry using the *Project to Surfaces tool* in the second step of meshing (see Figure 2B). To include the inner wall, the O-Grid function was used where the inner perimeters of the boxes were assigned to the interior wall (see Figure 2C). The *Pure-Hexa function* was then applied to generate a hexahedral structured mesh between the inner and outer geometries with the desired density. It is worth mentioning that a uniform thickness of the adventitial layer was considered in both healthy and diseased geometries which was incorporated in the meshing protocol by seeding the elements radially, i.e., the thickness of the outermost layer of elements in the vessel wall. Figure 2D presents the final mesh generated from a carotid bifurcation of a healthy volunteer.

**FIGURE 3:**
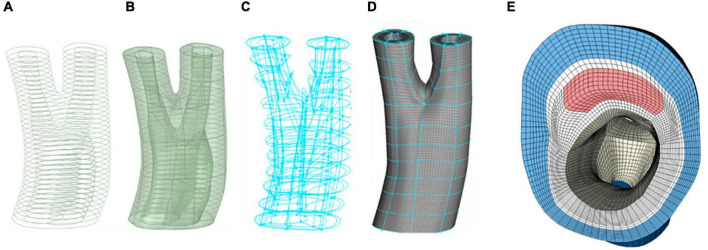
Different stages of meshing a diseased carotid with plaque atheroma and lipid pool. **(A)** Contours of the different components of the arterial wall. **(B)** Constructing and smoothing the inner and outer surfaces. **(C)** The connectivity of the boxes associated with each component of the vessel wall. **(D)** Hexahedral meshing of the boxes. **(E)** A cross section of the common carotid artery indicating the components in the vessel wall and quality of the mesh at the apex of the bifurcation.

Figure 3 depicts the different stages of the meshing protocol applied to a geometry obtained from a patient with carotid artery disease under evaluation for an endarterectomy surgery.

### Constitutive Equations

#### Kinematics

Arterial tissue is most commonly considered as an almost incompressible material (Holzapfel et al., 2000). A multiplicative decomposition of the deformation gradient tensor **F** into volumetric $J^{\frac{1}{3}}\,\text{I}$ and isochoric parts $\bar{\text{F}}$ is usually performed to characterise the deformation in arterial walls. Following Flory (1961), multiplicative decomposition of the deformation gradient tensor can be presented as follows;

(1) $$\text{F}=\left(J^{\frac{1}{3}}\,\text{I}\right)\,\bar{\text{F}}$$

where **I** is the identity tensor. The right Cauchy-Green tensor, denoted by **C**, and its isochoric counterpart, denoted by $\bar{\text{C}}$, are then defined as follows;

(2) $$\text{C}={\text{F}}^{T}\,\text{F}=J^{\frac{2}{3}}\,\bar{\text{C}},\bar{\text{C}}={\bar{\text{F}}}^{T}\,\bar{\text{F}}$$

The deviatoric principal invariants of the right Cauchy-Green tensor can be written as follows.

(3) $$\begin{array}{l} {\bar{I}}_{1}=tr(\bar{C}),\\ {\bar{I}}_{2}=\frac{1}{2}[tr^{2}(\bar{C})-tr({\bar{C}}^{2})],\\ {\bar{I}}_{3}=\text{det}(\bar{C}) \end{array}$$

Direction of the fibres can be defined using a unit vector **M** in the undeformed configuration. The isochoric configuration of the vector **M** in the spatial coordinate system can then be written as follows;

(4) $$\bar{\text{M}}=\bar{\text{F}}\,\text{M}$$

The deviatoric invariants that associate with the unit vector **M** can be written as follows;

(5) $${\bar{I}}_{M}=t\,r\,\left(\bar{\text{C}}\,\bar{\text{M}}⊗\bar{\text{M}}\right)=\bar{\text{M}}.\left(\bar{\text{C}}\,\bar{\text{M}}\right)$$

#### Strain Energy Functions

In this study the anisotropic hyperelastic constitutive model, proposed in Gasser et al. (2006) was used to capture the mechanical behaviour of the healthy and diseased arterial wall in the healthy and diseased arteries (Gasser et al., 2006). This constitutive model captures the mechanical behaviour of the soft tissue by additively decomposing the response of the tissue into its components as follows;

(6) $$\psi =\psi_{v\,o\,l}+{\bar{\psi }}_{i\,s\,o}+{\bar{\psi }}_{c\,f}$$

The response of the non-collagenous tissue was captured using the neo-Hookean material model (${\bar{\psi }}_{i\,s\,o}$). The response of the collagenous tissue was captured by postulating two symmetric families of collagen fibres, ${\bar{\psi }}_{c\,f}$. The volumetric free energy function (ψ_*vol*_) can be expressed as follows;

(7) $$\psi_{v\,o\,l}\,\left(J\right)=\frac{1}{2}\,\kappa_{0}\,{\left(J-1\right)}^{2}$$

where κ_0_ serves as a penalty parameter that controls the compressibility of the biological soft tissue (Gasser and Holzapfel, 2002).

The isotropic strain energy function (SEF), (${\bar{\psi }}_{i\,s\,o}$), can be written as follows.

(8) $${\bar{\psi }}_{i\,s\,o}\,\left(\bar{\text{C}}\right)=\frac{1}{2}\,\mu \,\left({\bar{I}}_{1}-3\right)$$

where μ is the shear modulus of the ground matrix. The SEF to capture the mechanical behaviour of the collagenous tissue can be written as follows;

(9) $${\bar{\psi }}_{c\,f}\,\left(\bar{\text{C}}\right)=\sum_{M=M_{4},6}\frac{k_{1}}{2\,k_{2}}\,\left(\text{exp}\,\left(k_{2}\,{\left(\kappa \,{\bar{I}}_{1}+\left(1-3\,\kappa \right)\,{\bar{I}}_{M}-1\right)}^{2}\right)-1\right)$$

where *k*_1_ and *k*_2_ are material parameters and ${\bar{I}}_{M_{4}}$ and ${\bar{I}}_{M_{6}}$ are the square of the stretch in the direction of collagen fibres and correspond to two unit vectors, **M**_4_ and **M**_6_, respectively. For further information on this constitutive model the reader is referred to Gasser et al. (2006). Having these SEFs, Cauchy stress can then be defined as follows;

(10) $$\sigma =\frac{2}{J}\,\text{F}\,\frac{\partial \psi }{\partial \text{C}}\,{\text{F}}^{T}$$

#### Remodelling Algorithm

Collagen fibre directions evolve *in vivo* to maximise the load bearing capacity of the tissue. Following Hariton et al. (2007) and Fausten et al. (2016), it was assumed that fibres are located in the plane made by the eigenvectors of the two largest principal Cauchy stresses σ_1_ and σ_2_ (Hariton et al., 2007; Fausten et al., 2016). The spectral decomposition of this stress tensor can be written as follows;

(11) $$\,\sigma =\sigma_{1}\,{\vec{e}}_{1}⊗{\vec{e}}_{1}+\sigma_{2}\,{\vec{e}}_{2}⊗{\vec{e}}_{2}+\sigma_{3}\,{\vec{e}}_{3}⊗{\vec{e}}_{3}$$

where σ_1_≥σ_2_≥σ_3_. In the first step of this remodelling algorithm, the influence of collagen fibres on the response of the tissue was neglected. The stress values, in this step, were calculated using a neo-Hookean material model. This assumption was also made in other remodelling algorithms (Driessen et al., 2005b; Creane et al., 2011). One motivation behind such an assumption is that the anisotropic behaviour of soft tissue develops as a result of tissue remodelling and adaptation to mechanical loads and the mechanical behaviour of the tissue at neonatal stage is isotropic (Driessen et al., 2003, Driessen et al., 2005b; Kuhl et al., 2005; Hariton et al., 2007).

The ratio between the magnitude of the two largest principal stresses was used to define the angle of alignment of fibres with respect to the direction of the maximum principal stress, as follows;

(12) $$t\,a\,n\,\left(\alpha \right)=\sigma_{2}/\sigma_{1}$$

Using this equation, two-unit vectors defining the optimum directions of two families of collagen fibres in the spatial configuration were obtained as follows;

(13) $$\vec{{\text{m}}_{4\,o\,p}}=\cos\left(\alpha \right)\,{\vec{e}}_{1}+\sin\left(\alpha \right)\,{\vec{e}}_{2}$$

(14) $$\vec{{\text{m}}_{6\,o\,p}}=\cos\left(\alpha \right)\,{\vec{e}}_{1}-\sin\left(\alpha \right)\,{\vec{e}}_{2}$$

To calculate the stresses in the current configuration, the distribution of fibres in the undeformed configuration needed to be determined. For this purpose, the vectors $\vec{{\text{m}}_{4\,o\,p}}$ and $\vec{{\text{m}}_{6\,o\,p}}$ were pulled back to the reference configuration as follows;

(15) $$\vec{{\text{M}}_{4\,o\,p}}=\frac{{\text{F}}^{-1}\,\vec{{\text{m}}_{4\,o\,p}}}{\left|{\text{F}}^{-1}\,\vec{{\text{m}}_{4\,o\,p}}\right|}$$

(16) $$\vec{{\text{M}}_{6\,o\,p}}=\frac{{\text{F}}^{-1}\,\vec{{\text{m}}_{6\,o\,p}}}{\left|{\text{F}}^{-1}\,\vec{{\text{m}}_{6\,o\,p}}\right|}$$

In this study, dispersion of fibres was also subjected to remodelling rules. Following Driessen et al. (2008), the ratio of the maximum and intermediate principal stresses was used to define the optimum dispersion of the fibres in the spatial configuration, as follows,

(17) $$b=\frac{\sigma_{1}}{\sigma_{2}}-1$$

where b is the concentration parameter. This parameter can be attributed to the von-Mises periodic distribution function as follows.

(18) $$\rho \,\left(\alpha ,b\right)=4\,\sqrt{\frac{b}{2\,\pi }}\,\exp\left[b\,c\,o\,s\,\left(2\,\alpha \right)+1\right]/e\,r\,f\,i\,\left(\sqrt{2\,b}\right)$$

The von-Mises distribution function has been widely used to define the distribution of collagen fibres in arterial tissue (Gasser et al., 2006; Schriefl et al., 2012; Qi et al., 2015). The concentration parameter *b* was correlated with the dispersion parameter κ as follows;

(19) $$\kappa =\frac{1}{4}\,\int_{0}^{\pi }\rho \,\left(\alpha ,b\right)\,\sin^{3}\left(\alpha \right)\,\text{d}\,\left(\alpha \right)$$

**FIGURE 4:**
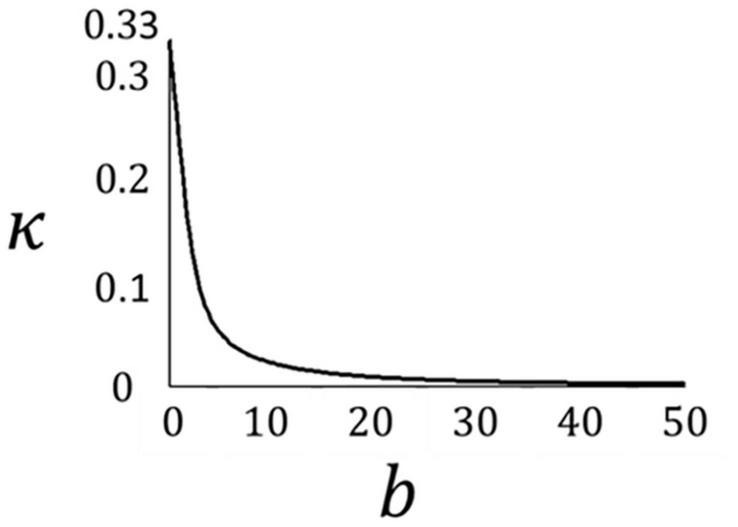
Dispersion- concentration parameter curve obtained from Equation 19.

Figure 4 presents the relationship between the concentration parameter *b* and the dispersion parameter κ. This relationship was obtained using Equation 19. For further information on the von Mises distribution function and the relationship between dispersion and the concentration parameter, the reader is referred to Gasser et al. (2006).

Using Equations (17)–(19), the optimum dispersion parameter was calculated in the spatial configuration. Knowing the optimum vectors representing each family of the fibres (Equations 15 and 16) and the optimum dispersion of fibres, the preferred distribution of fibres in the deformed configuration can be presented using the concept of generalised structure tensors as follows;

(20) $$\text{h}=c\,\text{I}+\left(1-3\,c\right)\,\vec{\text{a}}⊗\vec{\text{a}}$$

**FIGURE 5:**
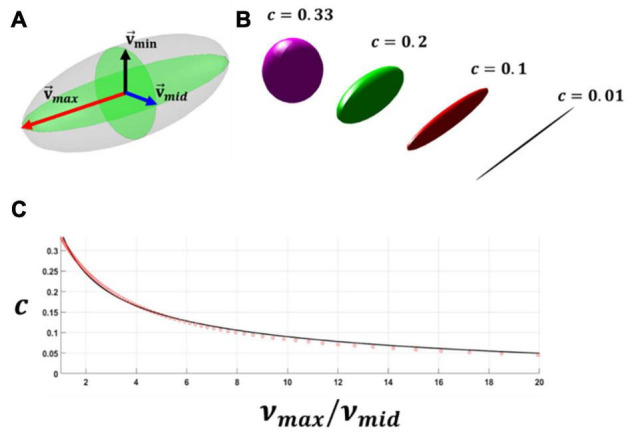
**(A)** A schematic representation of each family of the collagen fibres in an ellipsoid form using the eigenvalues and eigenvectors of the generalised structure tensor h. **(B)** A schematic representation showing the dispersion of the fibres and eccentricity of the ellipsoid. **(C)** The figure relating the ratio of the maximum and intermediate eigenvalues of the generalised structure tensor to dispersion of the fibres.

Were $\vec{\text{a}}$ is an arbitrary vector presenting one family of fibres and *c* is the dispersion of fibres. The symmetric second-order tensor **h** can be visualised using an ellipsoid where eigenvalues (*v*_*max*_,*v*_*mid*_,*v*_*min*_) and eigenvectors (${\vec{\text{v}}}_{m\,a\,x},{\vec{\text{v}}}_{m\,i\,d},{\vec{\text{v}}}_{m\,i\,n}$) of this tensor represent the direction and eccentricity of the ellipsoid (see Figure 5). In this study, the dispersion parameter *c* is correlated to the ratio of the two largest eigenvalues (ν_*max*_/ν_*mid*_) of the structure tensor **h**. For this purpose, the dispersion parameter *c* was calculated at different ratios of ν_*max*_/ν_*mid*_ using Equation 20. Then the relationship between the ratio of the two largest principal eigenvalues and dispersion parameter *c* was calculated by performing curve fitting (Equation 21, see Figure 5). Doing this, the eccentricity of this ellipsoid was correlated to the dispersion parameter *c*. It should be mentioned that once the ratio of ν_*max*_/ν_*mid*_ exceeded the magnitude of 20 the distribution of fibres was assumed to be fully anisotropic and *c* was assumed to be equal to 0.01, this is also demonstrated in Figure 5.

(21) $$c=0.421*{\left(\frac{\nu_{m\,a\,x}}{\nu_{m\,i\,d}}\right)}^{-0.4154}-0.0717$$

To calculate the optimum dispersion of fibres in the undeformed configuration a generalised structure tensor was initially made in the deformed configuration using both the optimum fibre direction and dispersion of fibres. This structure tensor **h** was then pulled back to the undeformed configuration as follows;

(22) $$\text{H}={\text{F}}^{-1}\,{\text{hF}}^{-T}$$

The spectral decomposition of the tensor **H** was calculated to determine the eigenvalues of the structure tensor **H** in the undeformed configuration. The ratio of the maximum and intermediate eigenvalues in the undeformed configuration was then used to calculate the dispersion of fibres in the undeformed configuration, using Equation 21.

**FIGURE 6:**
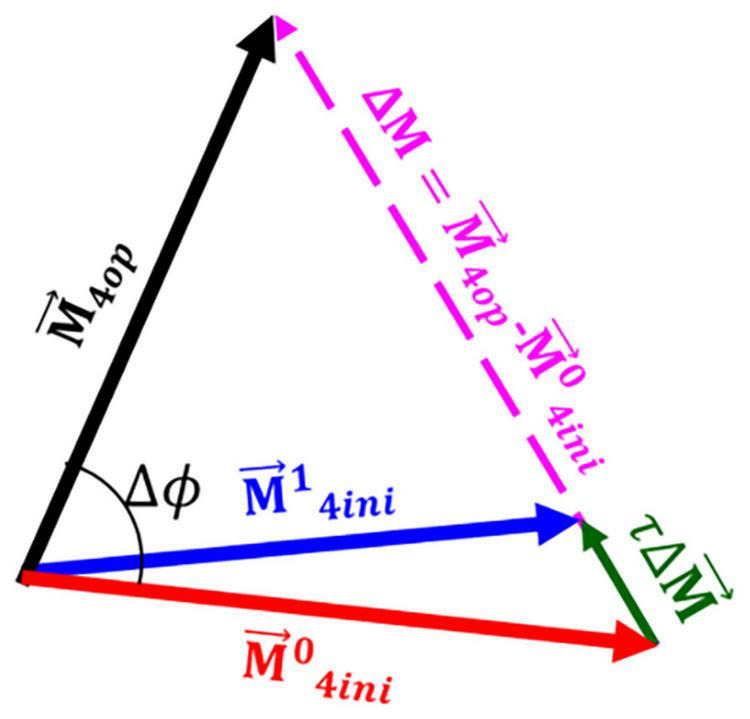
A schematic presentation of the remodelling process. Once the vector representing the optimum direction of the fibres was calculated $\left({\vec{M}}_{4\,o\,p}\right)$ a fraction of the total difference between the optimum fibre configuration and the initial configuration ($\tau \,\Delta \,\vec{M}$) was added to the initial configuration of the fibres $\left({\vec{M}}_{4\,i\,n\,i}^{0}\right)$ resulting in the initial configuration for the next iteration of the remodelling process $\left({\vec{M}}_{4\,i\,n\,i}^{1}\right)$. In this figure, Δϕ indicates the angle between the optimum fibre direction and initial direction of fibres.

Once the optimum direction and dispersion of fibres was calculated, both vectors and dispersion of fibres remodelled toward this preferred configuration. The vector of fibres was remodelled incrementally in the undeformed configuration toward its optimum direction by adding a fraction (τ) of the total difference between the optimum direction and initial direction of fibres. This process also established the next initial direction of the fibres for the next remodelling step (see Figure 6). A similar approach was used in Fausten et al. (2016).

The dispersion of fibres was remodelled toward its optimum value using a linear rate equation as follows;

(23) $$\frac{d\,\kappa }{d\,t}=\frac{1}{\tau_{\kappa }}\,\left(\Delta \,\kappa \right)=\frac{1}{\tau_{\kappa }}\,\left(\kappa -\kappa^{i\,n\,i}\right)$$

#### Remodelling Metric

The stiffness of arterial tissue has been associated with the orientation of fibres in studies such as Holzapfel et al. (2000) and Gasser et al. (2006). In this study, the concept of CDM was employed to correspond the stiffness of the arterial tissue to the distribution of fibres. Arterial tissue is assumed to have its optimum stiffness where collagen fibres are aligned with the predicted optimum configuration of fibres. Any deviation from this optimum configuration results in softening and weakening of the tissue. Here, we used two internal variables (γ_*RM*_ and β_*RM*_) to capture the softening that is associated with the deviation of fibres from their optimum configuration. This remodelling metric can then be used as a metric for assessing the vulnerability of atherosclerotic plaque in the arterial tissue.

Evolution of the internal variable γ during the remodelling process ([0,T]) can be written as follows;

(24) $$\gamma_{R\,M}=\max_{t\in \left[0,T\right]}\left\langle {\bar{I}}_{M}^{*}-{\bar{I}}_{M}^{*i\,n\,i}\right\rangle$$

Where ${\bar{I}}_{M}^{*}$ is a pseudo invariant associated with each family of fibres (**M**) and can be written as follows;

(25) $${\bar{I}}_{M}^{*}=\kappa \,{\bar{I}}_{1}+\left(1-3\,\kappa \right)\,{\bar{I}}_{M}$$

In Equation (24), ${\bar{I}}_{M}^{*i\,n\,i}$ is the value of ${\bar{I}}_{M}^{*}$ defined at the beginning of the remodelling process. The internal variable β_*RM*_ can be defined as follows;

(26) $$\beta_{R\,M}=\left\langle {\tilde{\beta }}_{R\,M}-{\tilde{\beta }}_{R\,M}^{i\,n\,i}\right\rangle$$

where ${\tilde{\beta }}_{R\,M}^{i\,n\,i}$ is the value of the variable ${\tilde{\beta }}_{R\,M}$ at the initial increment of the remodelling step in the arterial tissue. The variable ${\tilde{\beta }}_{R\,M}$ can be written as follows;

(27) $${\tilde{\beta }}_{R\,M}=\int_{0}^{T}\left\langle {\bar{I}}_{M}^{*}\right\rangle \,ds$$

In Equations (24), (26), and (27), ⟨()⟩ are Macaulay brackets to filter out the negative values. A similar form for evolution of internal variables was used in studies such as Miehe (1996), Balzani et al. (2012), and Ghasemi et al. (2018).

These two internal variables were then attributed to a softening function as follows;

(28) $$R\,M=R\,M_{\infty }\,\left[1-\exp\left(-\frac{\gamma }{\gamma_{\infty }}\right)\right]\,\left[1-\exp\left(-\frac{\beta }{\beta_{s}}\right)\right],R\,M\in \left[0,1\right)$$

Where, γ_∞_ and β_*s*_ are material properties. *RM*_∞_ denotes a predefined maximum softening level for this function (*RM*_∞_ = 0.99).

#### Finite Element Implementation

This remodelling algorithm was implemented into the commercial FE package Abaqus (Dassault Systèmes Simulia corporations, Vèlizy-Villacoublay, France). A user subroutine (UMAT) was used to define the behaviour of different components of healthy and diseased arteries where the definition of the Cauchy stress and tangent modulus was required.

The tangent modulus was calculated computationally using a technique introduced by Miehe (1996) and used in Ghasemi et al. (2018).

The geometries were segmented into two parts: healthy arterial wall and plaque burden. Healthy vessel wall was composed of the media and adventitia layers and plaque burden was composed of plaque atheroma and lipid pool, where observed in the MRI images. Material properties of each component along with the references they were obtained from are shown in Table 2.

**TABLE 2:** Material properties used to characterise the mechanical behaviour of the healthy arterial wall and plaque burden in the carotid bifurcations.

	*C*_*10*_ [*kPa*]	*k*_1_ [*kPa*]	*k*_2_ [−]	γ_∞_ [*kPa*]	*β*_s_	
Media	67.28	20.6	18.8	−	−	Sommer et al., 2010
Adventitia	37.25	61.2	32.8	−	−	Sommer et al., 2010
Plaque atheroma	37.5	2,029	25.2	6.52	0.37	Balzani et al., 2012; Chai et al., 2015
Lipid pool	50.537	−	−	−	−	Teng et al., 2015

Three healthy and five diseased bifurcations were analysed in this study. The analysis was performed in three steps. In the first step, the artery was subjected to axial displacements. The healthy bifurcations were subjected to three different levels of axial strains of 0, 5, and 10%. However, the diseased bifurcations were subjected to 5% axial strain following Balzani et al. (2012).

In the second step of this simulation both healthy and diseased bifurcations were subjected to systolic blood pressure of 16 kPa (Paritala et al., 2018). Optimum vector and dispersion of fibres were calculated in the end of this step.

In the third step of this simulation both the direction vector and dispersion of fibres were subjected to remodelling rules. In the healthy arterial wall, for both the healthy and diseased bifurcation models, collagen fibres were assumed to be at 45^*o*^ with respect to the direction of the maximum principal stresses initially. Dispersion of fibres was also assumed to be 0.33 in the first increment of the remodelling step. To analyse the diseased tissue, three different cases were postulated as the initial configuration of fibres in the plaque tissue, where fibres were oriented: (i) parallel to the direction of intermediate principal stress, (ii) at 45 degrees with respect to the direction of maximum principal stresses, and (iii) parallel to the direction of the maximum principal stress.

## Results

### Remodelling in Healthy Carotid Arteries

**FIGURE 7:**
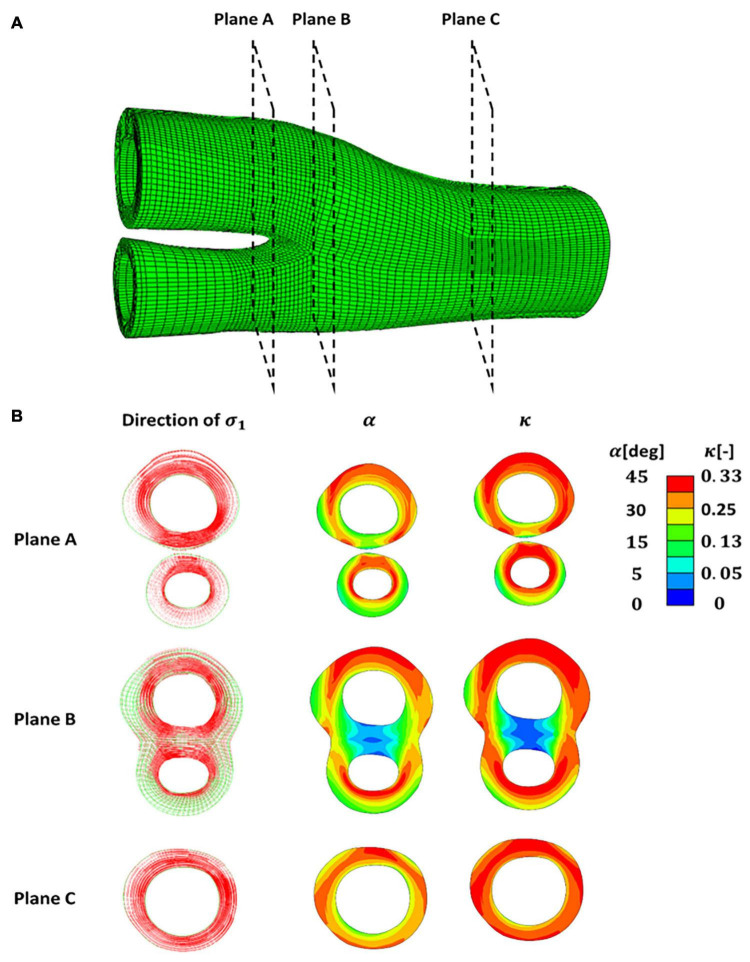
**(A)** A healthy carotid bifurcation cross sectioned using planes **(A–C)**. **(B)** Presentation of the directions of the maximum and intermediate principal stresses in each cross section along with the angle and dispersion of fibres. The angle of fibres (**α)** is presented with respect to the direction of the maximum principal stress.

In this part of the study, the distribution of fibres in the healthy carotid bifurcations was investigated. The direction of the max principal stresses in 3 different sections of a healthy carotid bifurcation under 5% axial strain are shown in Figure 7. The cross -section made by plane A shows the values obtained for angle and dispersion of fibres in the internal and external branches of this carotid bifurcation. Plane B and C correspond to the apex and common carotid artery. It is worth mentioning again that α represents the angle of fibres with respect to the direction of the maximum principal stress.

### Remodelling in Patient Specific Diseased Carotid Arteries

**FIGURE 8:**
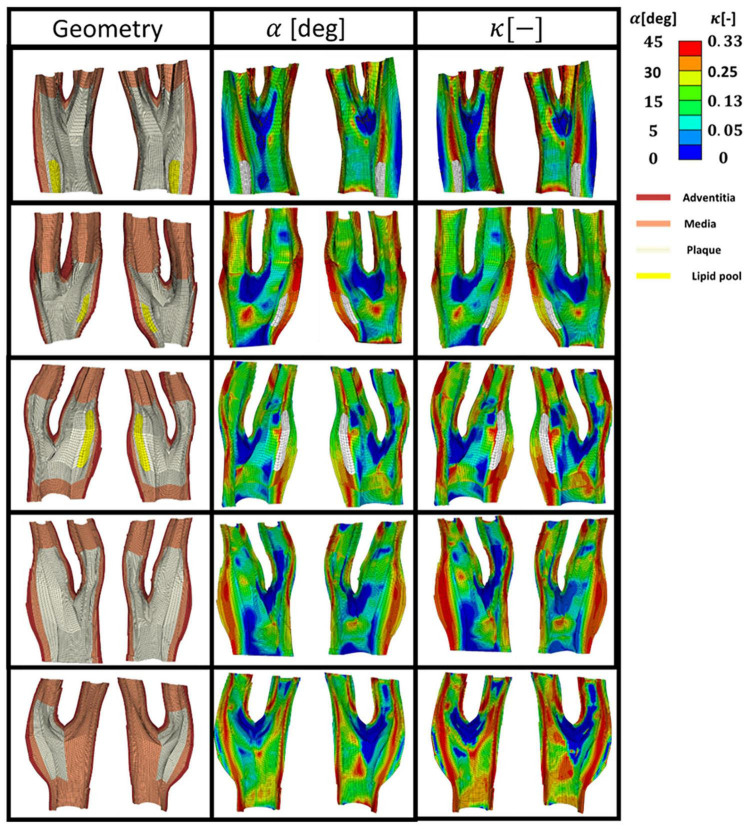
The geometry of the five diseased carotid bifurcations obtained from symptomatic patients consisting of the adventitia and media layers (healthy arterial wall) and plaque burden made of plaque atheroma and lipid pool. The predicted angle of fibres with respect to the direction of the maximum principal stress is shown in the second column. The corresponding dispersion of the fibres in each family of collagen fibres is shown in the third column.

Figure 8 presents five diseased bifurcations composed of healthy arterial wall and plaque burden. The healthy arterial wall is composed of the media and adventitia layers. The plaque burden is composed of plaque atheroma and lipid pool (where observed in the MRI images). The angle and dispersion of collagen is predicted in these geometries when the arteries are under 5% axial strain and the blood pressure is 16 kPa.

**FIGURE 9:**
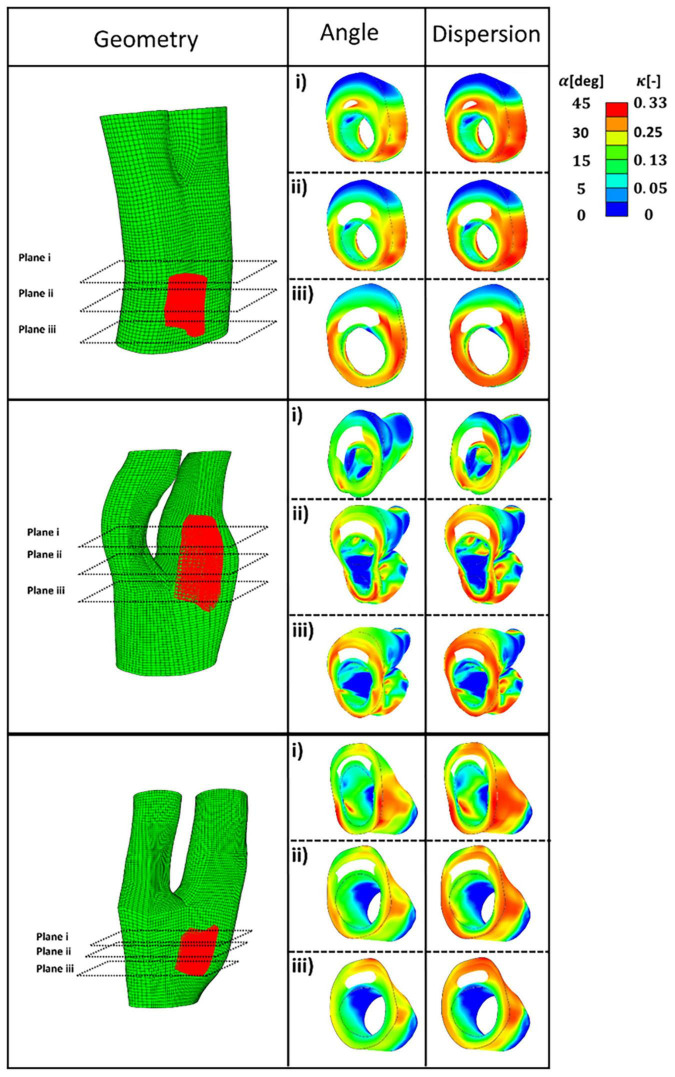
The predicted angle and dispersion of fibres in three cross-sections of diseased bifurcations where a lipid pool was observed in the geometry.

Figure 9 presents the values of angle and dispersion of fibres shown in Figure 8 in the cross-sections of the arterial walls where a lipid pool was observed in the geometry.

**FIGURE 10:**
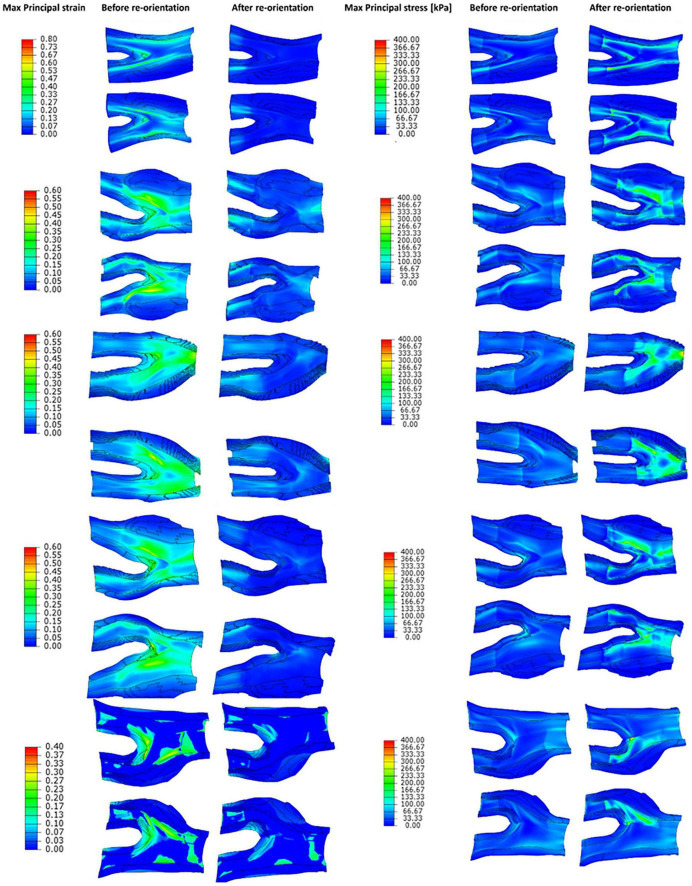
The contour plots of maximum principal strain and stress values obtained before and after remodelling of collagen fibres in the diseased geometries.

Strain and stress counter plots of diseased geometries before and after remodelling of collagen fibres toward the optimum fibre distribution are depicted in Figure 10. These geometries are ordered similar to Figure 8.

**FIGURE 11:**
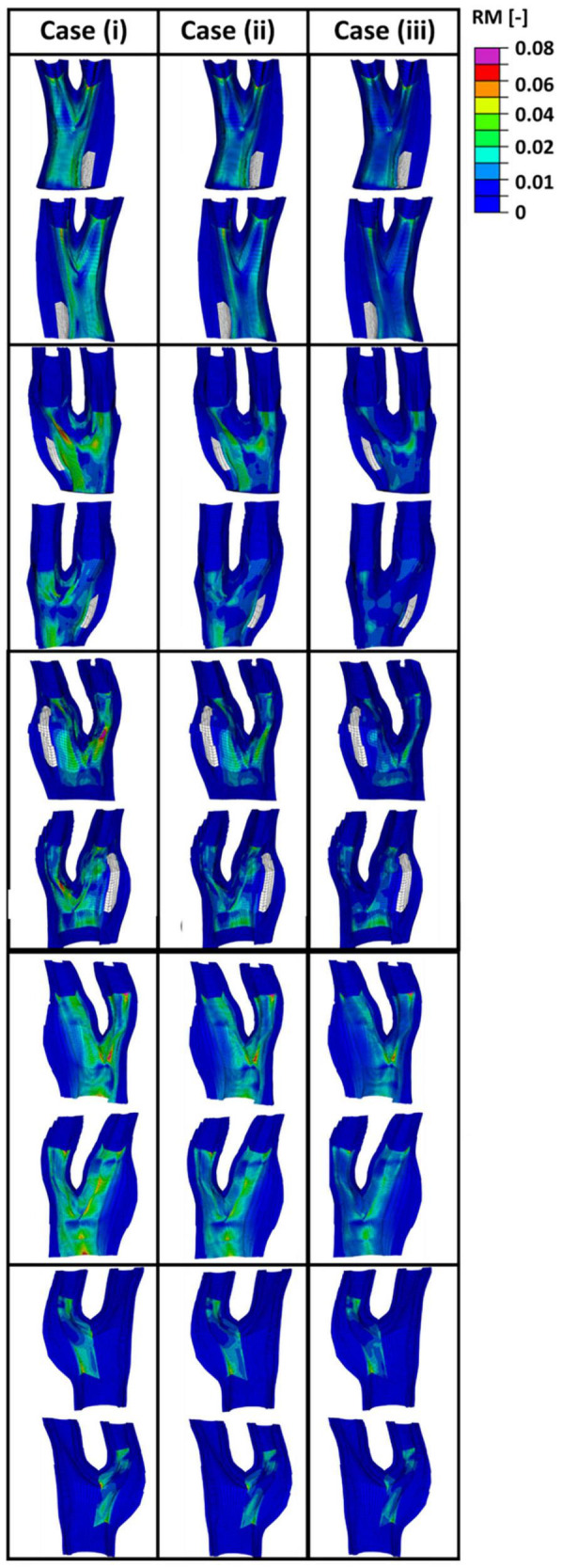
The values obtained for the remodelling metric (RM) during the remodelling process.

Figure 11 presents the values of the remodelling metric captured during the remodelling process in the diseased bifurcations by postulating the initial configuration of the fibres to be (i) parallel with the direction of intermediate principal stress (ii) at 45^*o*^ with respect to the direction of the maximum principal stress and (iii) parallel to the direction of the maximum principal stress. In each case collagen fibres re-orientated toward the optimum distribution calculated according to the ratio of max to intermediate principal stresses. Evolution of the internal variables during the remodelling process was used as an indicator for the lack of remodelling. It is also assumed that there is no lack of remodelling in the healthy arterial walls. It can be seen from this figure that the remodelling metric has the highest values in case (i) where fibres had the largest remodelling gap from the optimum distribution.

**FIGURE 12:**
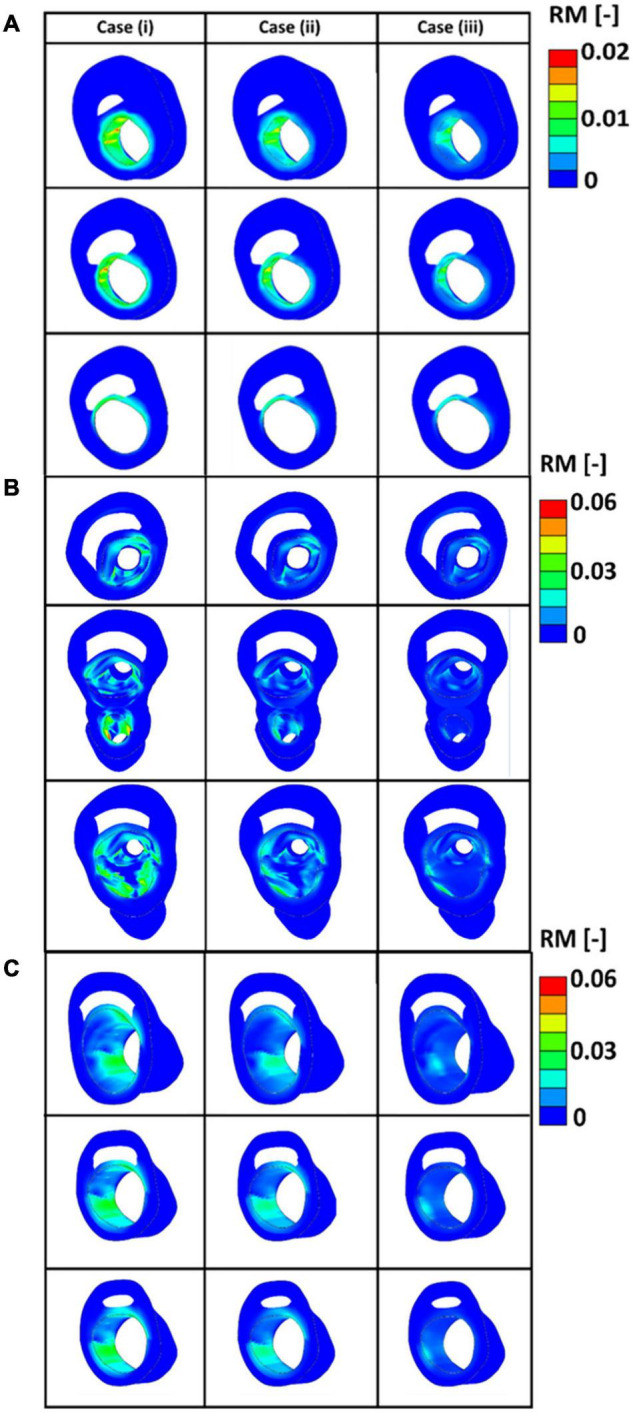
**(A)** The values of the remodelling metric for the three different cases at cross sections of the diseased bifurcation corresponding to the first plane of Figure 9. **(B)** The values of the remodelling metric for the three different cases at cross sections of the diseased bifurcation corresponding to the second plane of Figure 9. **(C)** The values of the remodelling metric for the three different cases at cross sections of the diseased bifurcation corresponding to the third plane of Figure 9.

Figure 12 presents the values obtained for the remodelling metric at three different cross sections of the diseased bifurcations. Figure 12A presents the cross sections that corresponds to the bifurcation depicted by plane 1 of Figure 9. The cross-sections from arteries in plane 2 and plane 3 of Figure 9 are shown in Figures 12B,C, respectively. It can be seen from these figures that arteries which correspond to case (i), where fibres were parallel with the direction of the intermediate principal stress, show highest values for the remodelling metric. This indicates that in these areas there is a higher need for remodelling.

## Discussion

Distribution of collagen fibres evolve *in vivo* to maximise the load bearing capacity of the arterial tissue. Collagen fibres grow and remodel during a person’s lifetime to contribute to bearing the load imposed by blood pressure. As a result, fibres in the media layer grow more in the circumferential directions, the direction of the max principal stress, while the distribution of the fibres remains almost isotropic in the adventitial layer (Krasny et al., 2017).

In this study, the distribution of collagen fibres in healthy and diseased carotid bifurcations were predicted. Both healthy and diseased geometries were meshed using hexahedral elements. There are many reasons why structured hexahedral meshes should be preferred when it comes to FE analysis of arterial tissue. Arterial tissue acts as a non-linear, nearly incompressible material. Tetrahedral elements are known to cause severe locking issues when it comes to dealing with such constitutive laws (Puso and Solberg, 2006). Using a structured hexahedral mesh is particularly of importance when accurate stress measurement is required as using an unstructured tetrahedral mesh can result in obtaining higher values of stress due to their high stiffness (De Santis et al., 2011). Structured hexahedral meshes are also computationally more efficient when it comes to large complex models such as human carotid arteries (Creane et al., 2010). Here, a novel meshing technique was proposed that can be efficiently applied to produce high quality structured hexahedral meshes for carotid bifurcations considering both luminal and adventitial layers. It is worth mentioning that in former studies only the luminal surface of the arteries was used to generate hexahedral meshes for arterial walls (Creane et al., 2010). These luminal surfaces were then expanded radially to estimate the outer layers of the arteries. Such an approximation of the outermost layers of the arteries can have considerable influence when it comes to FE analysis of diseased carotid arteries where the presence of the plaque can result in outward remodelling of the arterial wall. It is also worth mentioning again that, here, the healthy arterial wall was segmented into the media and adventitia layers and plaque burden was segmented into plaque atheroma and lipid pool.

Having these geometries meshed, the angle and dispersion of the collagen fibres in the healthy carotid bifurcations were predicted first (see Figure 7 and Supplementary Figure 2). The distribution predicted for collagen fibres in the healthy carotid bifurcation agrees with experimental studies such as Chai et al. (2015), where MR-DTI technique was applied to characterise the distribution of the fibres and also where polarised light microscopy was used (Finlay et al., 1995). These results are also compatible with the result of the remodelling algorithm suggested by Creane et al. (2011) where a strain based remodelling algorithm was applied to capture both angle and dispersion of the fibres in the healthy vessel wall (Creane et al., 2011).

While many studies apply isotropic material models to characterise the mechanical behaviour of the atherosclerotic plaque in the carotid arteries (Creane et al., 2011; Teng et al., 2015), recently, experimental studies have revealed the highly anisotropic response of the atherosclerotic plaque in the carotid arteries (Chai et al., 2014; Johnston et al., 2021). Akyildiz et al. (2017) used DTI to investigate the distribution of the fibres in plaques. They observed varying distributions of the fibres in different regions of the carotid plaques (Akyildiz et al., 2017). Their overall measurement of the fibre orientation in the plaque showed that on average 52% of the fibres were in the circumferential direction of the arterial wall, 34% were in the longitudinal direction and 14% were orientated in the radial direction (Akyildiz et al., 2017). Schmidt et al. (2015) also reported that collagen fibres are more aligned with the axial direction in the plaque cap while in the fibrotic media fibres are more aligned in the circumferential direction of the vessel wall (Schmidt et al., 2015). Douglas et al. (2017) used histological techniques to explore the alignment of the fibres in coronary atherosclerotic plaques. In that study they observed that in the shoulder areas fibre alignment is reduced significantly compared to regions of the fibrous cap. However, they could not link the structure of the plaque to their observations from their FE simulation (Douglas et al., 2017). One reason behind such incompatibility between histological measurement and FE simulation can be explained by investigating the configuration in which fibres are studied. Using histological images to characterise the distribution of fibres can result in neglecting the role that loads such as blood pressure and axial stretch play to maintain the distribution of fibres in the arterial wall. In fact, histological techniques can only provide information about the distribution of the fibres in the unloaded configurations. This absence of loads can result in the re-orientation of the fibres and consequently discrepancies between the measured fibres in histology and those in an *in vivo* configuration. In addition, only information from the in-slice plane is obtained using histology and out of plane fibres are missed.

These variations in the distribution of the collagen fibres in the atherosclerotic plaque may be explained by looking at remodelling in diseased tissues. Bennett (1999) demonstrated that apoptosis, or cell death, can change the architecture of the arterial wall and lead to positive or negative remodelling of the artery during atherosclerotic plaque development (Bennett, 1999). Bennett (1999) also showed that apoptosis of the vascular smooth muscle cells (VSMCs) can be a major contributor to vulnerable atherosclerotic plaque rupture, where a paucity of VSMCs was observed particularly at plaque shoulders (Bennett, 1999). A paucity of VSMCs in the atherosclerotic atheroma can result in lack of remodelling as new collagen fibres cannot be produced in the preferred direction to maximise the load bearing capacity of the tissue. Such lack of remodelling can lead to weakening of the arterial wall and potentially result in plaque rupture.

In this study, the optimum distribution of collagen fibres which gives the maximum strength to the atherosclerotic plaque was predicted (see Figures 8, 9). The proposed remodelling algorithm showed that a highly aligned distribution of the fibres is required to withstand the loads of the blood pressure at the regions of the plaque shoulder. The proposed remodelling algorithm also predicted a more aligned distribution of fibres at regions of the fibrous cap (area of tissue between the lumen and lipid pool) compared to other regions of that particular cross section (see Figure 9). However, collagen fibres were predicted to be less aligned in the regions of the plaque cap compared to the plaque shoulders. This is explained by the fact that the plaque cap has a smaller cross sectional area compared to other areas of the atherosclerotic plaque, inducing higher stresses in the axial direction which results in larger ratios of the intermediate to max principal stresses.

The values obtained for max principal strain and stress before and after the remodelling process in the diseased geometries are shown in Figure 10. This figure shows that because of the remodelling of fibres toward the optimum fibre configuration the values of the maximum principal strains decrease in the arterial wall. This contraction of the tissue improves the integrity of the vessel wall, makes the plaque more stable and minimises the risk of plaque rupture. Also, this figure shows that the values of the stress in the arterial tissue increase which is due to the contribution of the fibres in bearing the loads. The other result of the contribution of fibres in bearing the load is the increase in the stiffness of the arterial walls. These observations explain the results achieved in studies such as Huang et al. (2016) where higher strain levels were found in plaques with higher levels of vulnerability (Huang et al., 2016).

In this study, a remodelling metric was also defined to characterise the lack of remodelling in the diseased carotid bifurcations to the optimum collagen fibre orientation. Motivated by the classification of alignment of fibres in carotid plaques performed in Akyildiz et al. (2017), three different initial configuration of the collagen fibres were postulated, case (i) where fibres were assumed to be parallel to the direction of the intermediate principal stress, case (ii) where fibres were assumed to be at 45^*o*^ degrees with respect to the direction of the maximum principal stresses and case (iii) where fibres were assumed to be parallel to the direction of the maximum principal stresses. In all cases the fibres were allowed to fully re-orient toward their optimum configuration. Evolution of the internal variables during the remodelling process was then used as an indicator for the lack of remodelling in the diseased atherosclerotic tissue (see Figure 12). The results indicated higher RM values at the areas of the plaque shoulders in case (i) where fibres were assumed to be parallel to the intermediate stress direction. This lack of remodelling to the optimum distribution could occur in diseased arteries due to a lack of cells present to produce fibres in the direction of the loads and/or possibly due to fibres being overly degraded (Gaul et al., 2018). This remodelling metric has the potential to be used as a pre-clinical indicator of the risk of atherosclerotic plaque rupture, particularly now that advancements in imaging modalities such as DTI show great promise in the *in vivo* characterisation of the structure of collagen fibres (Flamini et al., 2012; Akyildiz et al., 2017; Shahid et al., 2017; Tornifoglio et al., 2020).

Whilst this study offers a means to assess the role of fibre orientation in carotid plaque stability, there are some limitations to the study. Firstly, the geometries were obtained from the deformed configuration in the body and a blood pressure of 16 kPa was then applied on the arterial wall without pulling back the geometry to its unloaded configuration. The importance of calculating the unloaded state of the arterial wall is emphasised in studies such as Raghavan et al. (2006) and Chandra et al. (2016). The influence of the residual strains and stresses in the vessel walls was also not included. The importance of incorporating the residual stress in the mechanical behaviour of the arterial tissue is emphasised in studies (Delfino et al., 1997; Alastrué et al., 2007; Schröder and Brinkhues, 2014). The contribution of cells, which may play a very important role in the remodelling process, through production or degradation of the fibres or in the healing process, was also not incorporated in this study. Such limitations also exist in many other studies such as Hariton et al. (2007), Creane et al. (2010), and Fausten et al. (2016) and are the focus of future work in our group.

To the best of the author’s knowledge, however, this is the first time that a computational algorithm has been employed to predict the optimum distribution of collagen fibres in complex real diseased carotid bifurcations obtained from symptomatic patients and used to characterise the lack of remodelling therein. This study provides critical insights into the collagen fibre patterns required in carotid arterial and plaque tissue to maintain plaque stability.

## Data Availability Statement

The original contributions presented in the study are included in the article/Supplementary Material, further inquiries can be directed to the corresponding author/s.

## Ethics Statement

The studies involving human participants were reviewed and approved by St. James’s Hospital Dublin. The patients/participants provided their written informed consent to participate in this study.

## Author Contributions

MG: conceptualisation, methodology, formal analysis, investigation, and writing—review and editing. RJ: methodology and writing—review and editing. CL: conceptualisation, methodology, writing—review and editing, and supervision. All authors contributed to the article and approved the submitted version.

## Conflict of Interest

The authors declare that the research was conducted in the absence of any commercial or financial relationships that could be construed as a potential conflict of interest.

## Publisher’s Note

All claims expressed in this article are solely those of the authors and do not necessarily represent those of their affiliated organizations, or those of the publisher, the editors and the reviewers. Any product that may be evaluated in this article, or claim that may be made by its manufacturer, is not guaranteed or endorsed by the publisher.
