# Supplementary material for: Development of a Collagen Fibre Remodelling Rupture Risk Metric for Potentially Vulnerable Carotid Artery Atherosclerotic Plaques
Source: Front Physiol. 2021 Oct 29;12:718470. doi: 10.3389/fphys.2021.718470 (PMC8586512; doi:10.3389/fphys.2021.718470)
Supplement: Supplementary file 1 [file Data_Sheet_1.docx]

**Supplementary Material**

Supplement to: Ghasemi et al., **“ Development of a remodelling rupture risk metric for potentially vulnerable carotid atherosclerotic plaques”**

*Remodelling in an idealised cylindrical artery*

Figure S1 (A) and (B) present the values of $\alpha$ and $\kappa$ through an idealized arterial wall thickness as a result of different axial strains and blood pressure of 16 kPa. $\Delta\phi$ and $\Delta\kappa$ during the remodelling step are shown in Figure S1 (C) and (D), respectively. It can be seen that, the case with the lowest axial strain has the largest remodelling gap from the optimum distribution of fibres (Larger $\Delta\phi$ and $\Delta\kappa$). It should be noted again that the distribution of fibres in this simulation was assumed to be isotropic initially where fibres were postulated to be at ${45}^{o}$ with respect to the direction of max principal stresses and dispersion of fibres was assumed to be 0.33. Figure S1 (E) presents the decrease in the maximum principal strain in the healthy arterial wall as a response to the remodelling of fibres toward the maximum principal stress. It can be seen in this section that the samples with the largest remodelling gap experience the maximum decrease in the maximum principal strain. This section also indicates the contraction of the vessel wall as a result of the remodelling of fibres. Figure S1 (F) shows the changes in the maximum principal stresses during the remodelling of fibres toward their preferred distribution. This section indicates that although the maximum principal strain was decreased as a result of the remodelling of fibres toward the optimum distribution, the principal stress increased as a result of the cooperation of the collagen fibres in bearing the loads. It can also be seen that the sample with highest remodelling gap experience the maximum increase in the maximum principal stress. Figure S1 (G) presents the stretch in the sample in the direction of the collagen fibres during the remodelling process. This section also shows that collagen fibres in the sample with the largest remodelling gap will experience larger stretches to reach the optimum distribution. Figure S1 (H) shows the evolution of pseudo invariant $\bar{I}_{M}^{*}$, Equation 15, during the remodelling process. This section also shows that this invariant evolved to larger values in samples with larger $\Delta\Phi$ and $\Delta\kappa$. It should be mentioned that section C to H of Figure S1 present data obtained from the centroid of a luminal element while the medial and adventitial layers were meshed to have 8 and 4 elements respectively.


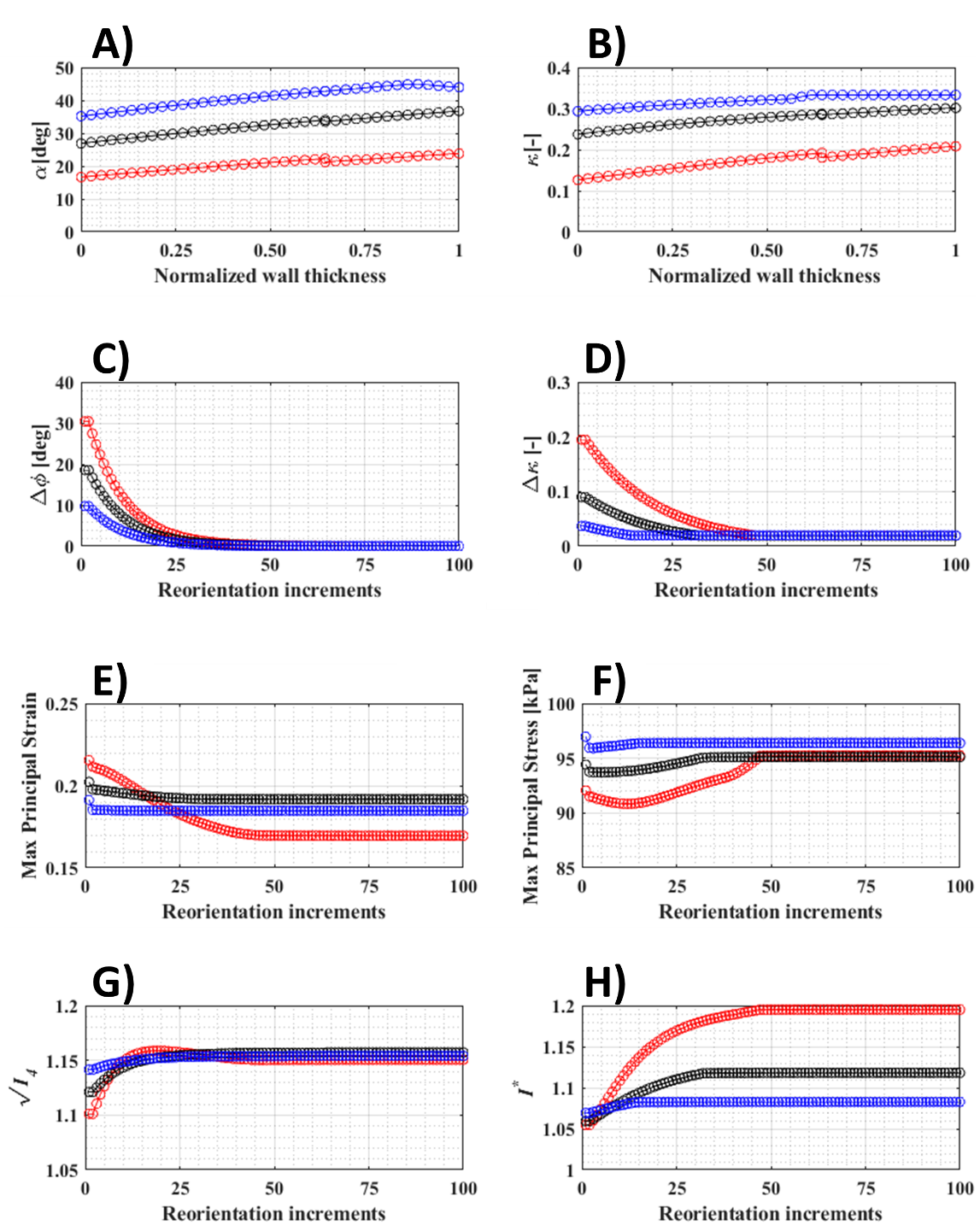


**Figure S1.** The influence of the reorientation of fibres on the mechanical behaviour of the arterial wall A) Angle of fibres with respect to the direction of the maximum principal stress through the wall thickness. B) Dispersion of the fibres through the normalized arterial wall thickness. Sections D to H presents values obtained from the centroid of the luminal element. C) The difference between the angle of fibres from the optimum direction of collagen fibres in the centroid of the luminal element during the remodelling step. D) The difference between the dispersion of fibres and the optimum distribution of fibres during the remodelling step in the centroid of the luminal element. Changes in the maximum principal stress and maximum principal strain during the remodelling step (E) and (F), respectively. G) Stretch in the sample in the direction of the collagen fibres. H) Evolution of the invariant $I^{*}$during the remodelling step.

*Observation of predicted angle and dispersion for three healthy bifurcations at different levels of axial strain*

Figure S2 presents geometries of three healthy carotid bifurcations. The predicted angle of fibres with respect to the direction of the maximum principal stress under three different levels of axial strain ($\varepsilon_{z}$) are shown in sections (I) to (III) for each geometry. The predicted dispersion of fibres in each family of collagen fibres is shown in the second row for each geometry, sections (IV) to (VI). This figure shows that in the absence of axial strain, collagen fibres are mainly predicted to be fully aligned with the direction of the maximum principal stress, with low angles and dispersion values. However, it can be seen that by increasing the axial strain, fibres re-orientate to maximize the load bearing capacity of the tissue in the plane made by directions of $\sigma_{1}$ and $\sigma_{2}$. It can also be observed that the distribution of the fibres remains constant in certain regions of healthy bifurcations although higher levels of axial strain were imposed on these arteries. The apex of the bifurcation in all three healthy geometries under different levels of axial strain exhibit highly aligned collagen fibre distribution. This concentrated distribution of fibres is the result of high values of maximum principal stress compared with the intermediate principal stresses in these regions. One main reason behind such large differences is the high curvature of the lumen at the apex which leads to high stress concentrations at these regions. It can also be observed that under the apex there are regions of near isotropic distribution of the fibres, regions with $\alpha={45}^{o}$ and $\kappa=0.333$. These observations agree with the results of studies such as Creane et al (2011) and Hariton et al. (2007) [28], [30].

The highly aligned orientation of the collagen fibres in the samples with no axial strain is shown in Figure S3 (A). The influence of the axial strain of 10% on the orientation of the fibres can be seen in Figure S3 (B). The highly aligned orientation of the fibres at the location of the apex is indicated in the top views using dashed rectangles.


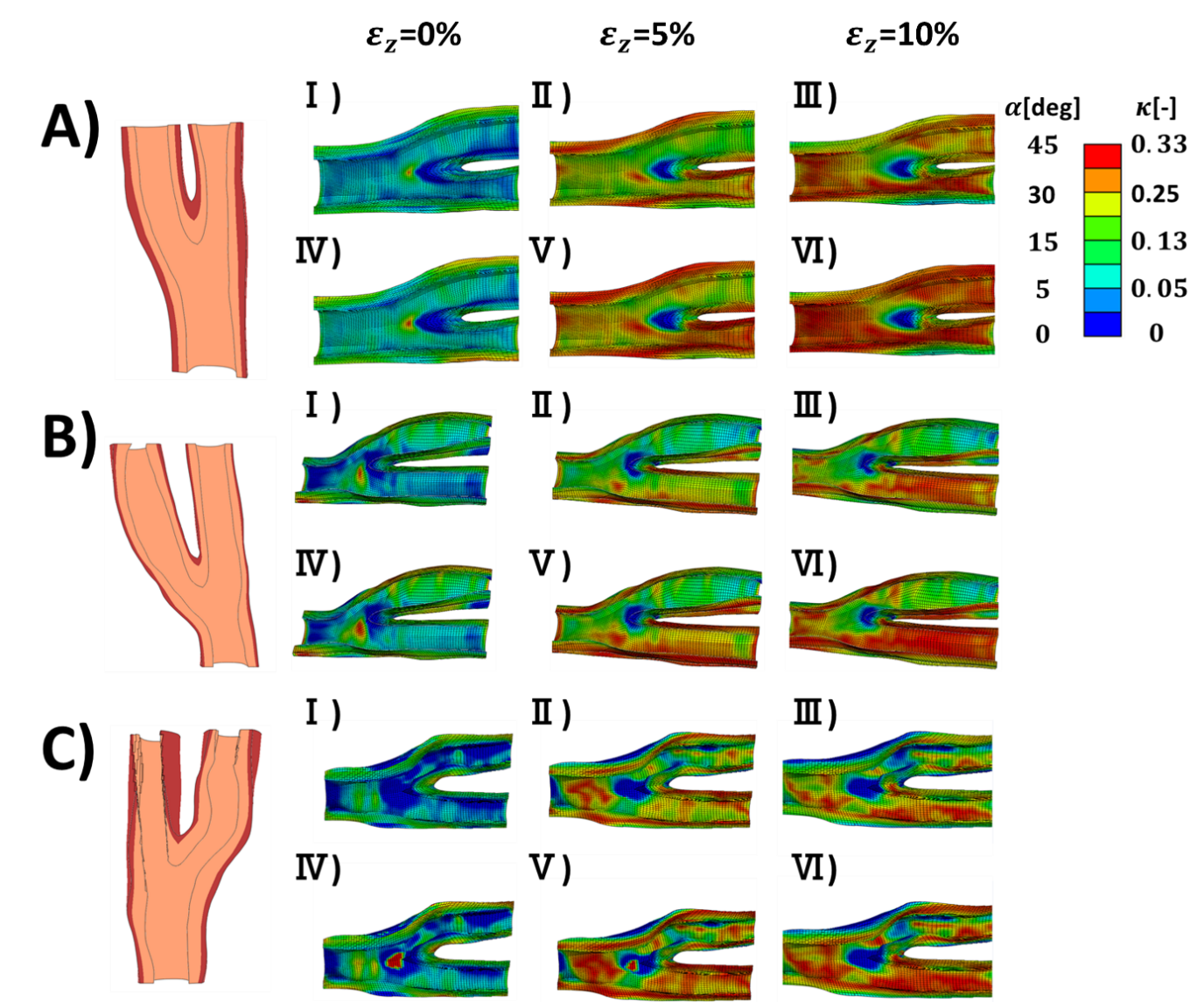


**Figure S2.** Predicted angle and dispersion of collagen fibres in three healthy bifurcations. The first row for each geometry indicates the angle of fibres and the second row for each geometry indicates dispersion of the fibres


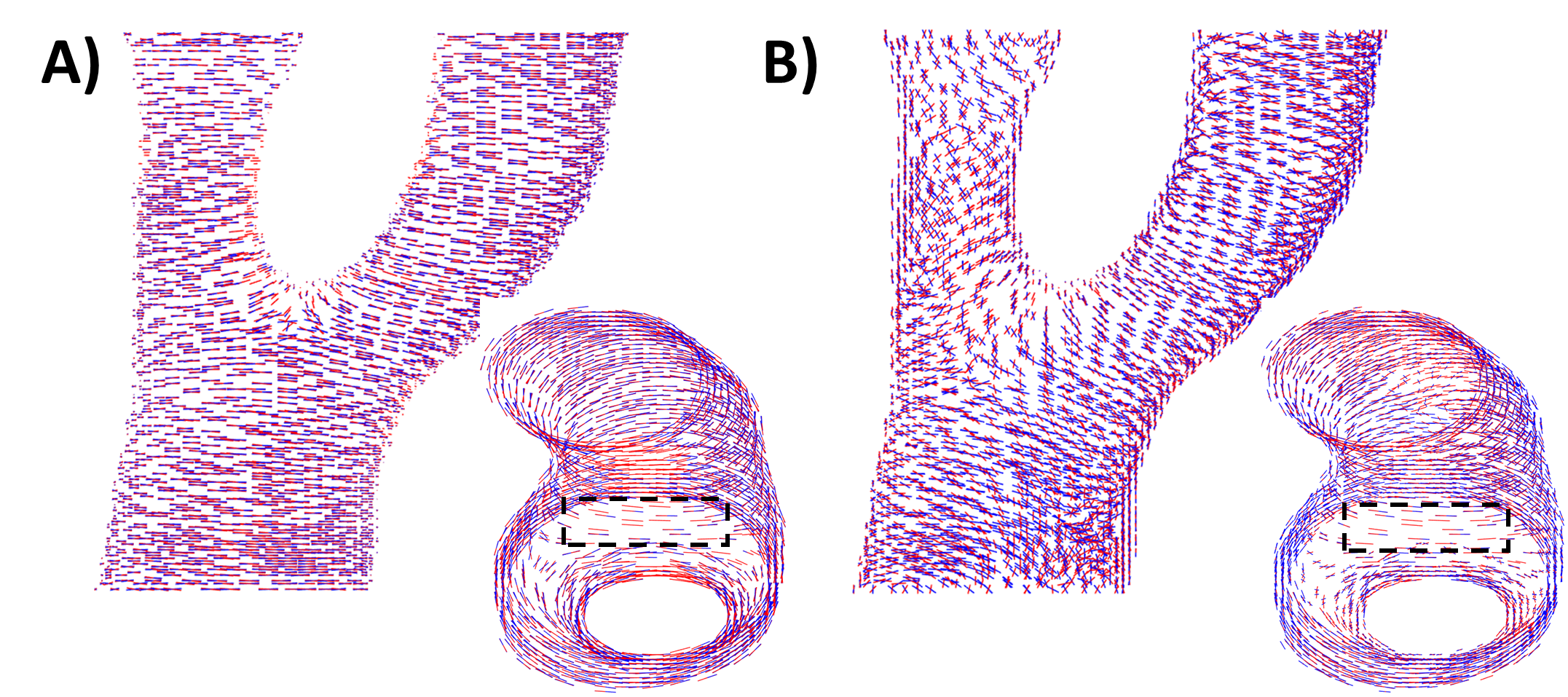


**Figure S3.** The orientation of the fibres A) without applied axial strain and B) with applied axial strain of 10% under blood pressure of the 16 kPa.

Three healthy carotid bifurcations were analysed at three different axial strain levels: 0, 5 and 10%. It was observed that in the absence of the axial stretch the distribution of the fibres was predicted to be highly anisotropic and aligned in the circumferential direction of the vessel wall. However, increasing the axial stretch resulted in a more isotropic distribution. The reason behind such isotropic distribution at 10% axial strain is that this loading condition imposes high axial stresses on the vessel wall and increases the ratio of the ($\sigma_{mid}/\sigma_{max}$).

.
